# Propensity score-adjusted analysis on early tirofiban administration to prevent thromboembolic complications during stand-alone coil embolization of ruptured aneurysms

**DOI:** 10.1038/s41598-024-77354-1

**Published:** 2024-11-01

**Authors:** Franziska Bürkle, Charlotte S. Weyland, Dimah Hasan, Farzaneh Yousefi, Hani Ridwan, Omid Nikoubashman, Martin Wiesmann

**Affiliations:** Department of Diagnostic and Interventional Neuroradiology, University Hospital RWTH Aachen, Aachen, Germany

**Keywords:** Tirofiban, Platelet aggregation inhibitors, Subarachnoid hemorrhage, Intracranial aneurysm, Heparin, Propensity score, Neurological disorders, Stroke

## Abstract

The glycoprotein IIb/IIIa antagonist tirofiban has been shown to prevent thromboembolic events during endovascular procedures, but the benefits and risks of its prophylactic early intraprocedural administration for stand-alone coil embolization of acutely ruptured aneurysms are still unclear. We conducted a retrospective single-center analysis of patients treated for aneurysmal subarachnoid hemorrhage with stand-alone coil embolization. Two study cohorts were compared according to the primary prophylactic antithrombotic medication during the procedure: patients receiving only intravenous heparin (HEP) versus patients receiving tirofiban in addition to heparin prior to final aneurysm obliteration (HEP + TF). Outcome endpoints were the incidence of angiographically visible thrombus formation or distal embolization, and the incidence of periprocedural intracranial hemorrhage (ICH). Of 204 cases, 159 were prophylactically treated with HEP and 45 with HEP + TF. Intraprocedural thromboembolic events were less frequent with HEP + TF before and after propensity score matching (PSM) (2.5% vs. 19.7%, *p* = 0.017). The incidence of ICH and symptomatic ICH did not differ between HEP + TF and HEP before and after PSM (20.5% vs. 30.7%, *p* = 0.29; and 5.1% vs. 4%, *p* = 0.88). Early intraprocedural tirofiban administration may be effective in preventing thromboembolic complications during stand-alone coil embolization of acutely ruptured aneurysms without increasing the risk of ICH.

**Supplementary Information:**

The online version contains supplementary material available at 10.1038/s41598-024-77354-1.

## Introduction

Thrombus formation leading to thromboembolism is a well-known risk in endovascular procedures. After aneurysm rupture, coil embolization involves an increased risk of thromboembolism, owing to the associated hypercoagulable state beyond the thrombogenic potential of the endovascular procedure itself^1,2^. Platelet antagonists such as aspirin, clopidogrel, or tirofiban have been shown to effectively reduce the risk of thrombus formation, and are widely used in elective endovascular procedures^3,4^. There has also been growing research interest in the effectiveness and safety of tirofiban administration in emergency settings, yielding promising results in the endovascular treatment of acute ischemic stroke and prophylactically in stent-assisted coiling or flow diverter therapy for ruptured aneurysms^5–7^. Tirofiban is a reversible and intravenous glycoprotein (GP) IIb/IIIa receptor antagonist that leads to extensive platelet aggregation inhibition even in non-responders to oral antiplatelet agents^8–10^. After discontinuation, normal hemostatic function is restored within a few hours^11^, which can be clinically advantageous in various (acute) settings. However, with regard to stand-alone coil embolization of ruptured aneurysms, i.e. coil embolization without adjunctive parent vessel stenting using stents or flow diverters^12^, there is no evidence as to whether the antithrombotic effect of prophylactic tirofiban justifies the concomitant bleeding risk, which is especially feared to be high with early intraprocedural administration. Therefore, we investigated the early primary prophylactic use of intravenous tirofiban in addition to heparin during stand-alone coiling of ruptured aneurysms with regard to the incidence as well as the extent of intraprocedural thromboembolic events, and secondarily, the associated risk of intracranial hemorrhage (ICH). To establish baseline balance in our patient sample, we performed an additional propensity score matching (PSM) analysis.

## Methods

### Study design and patient selection

**Fig. 1:**
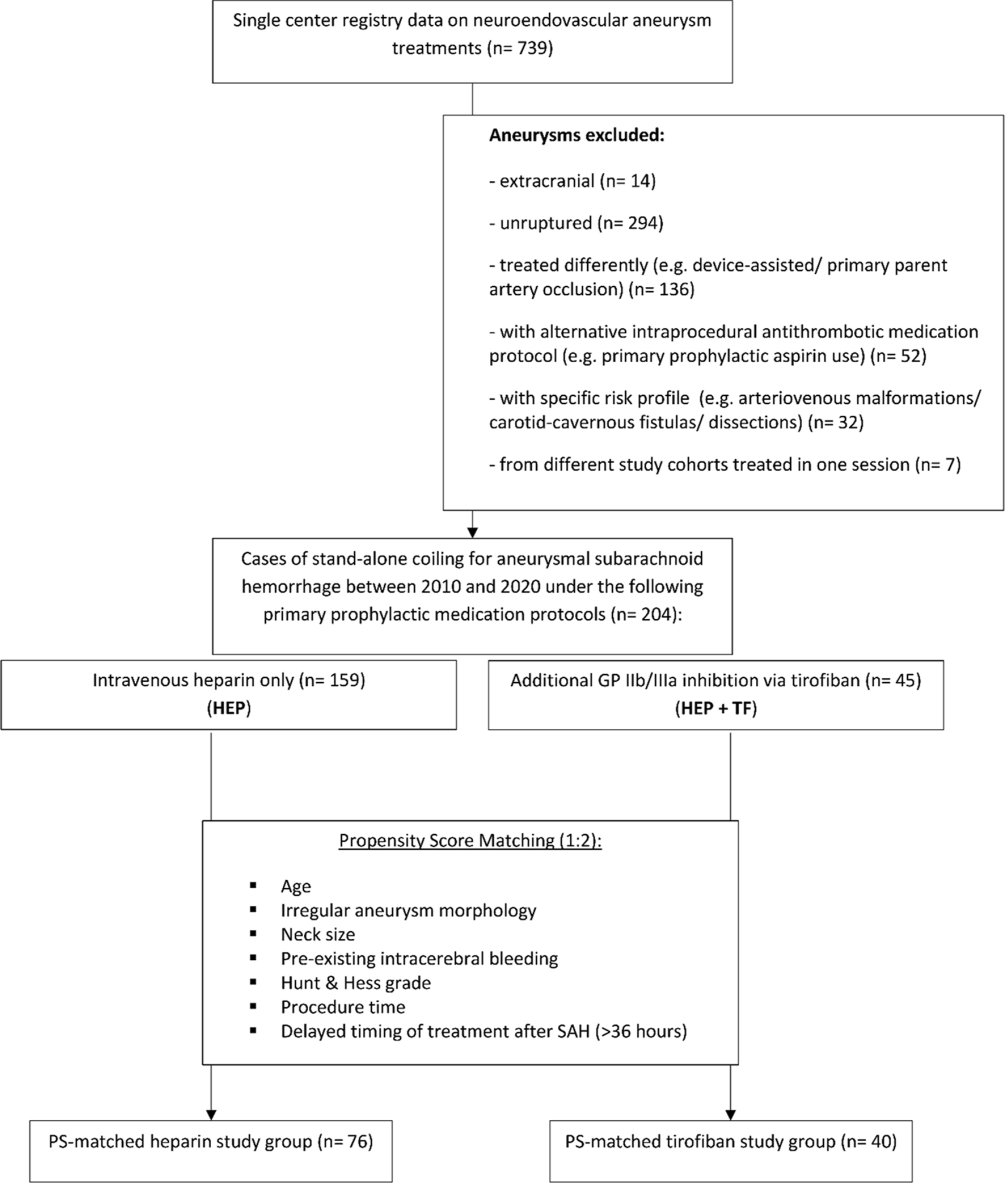
Flowchart of patient inclusion in the study. HEP, heparin; PS, propensity score; TF, tirofiban.

This retrospective data analysis was performed after approval from the local ethics board of our institution (Ethik-Kommission an der Medizinischen Fakultät der Rheinisch-Westfälischen Technischen Hochschule Aachen (RWTH Aachen), EK257-20). Due to the retrospective analysis of our study no experiments on humans and/or on human tissue samples were conducted. All methods were carried out in accordance with relevant guidelines and regulations. The need to obtain informed consent from patients was waived by our ethics board due to the retrospective nature of our analysis. All patients 18 years of age or older who were treated with stand-alone coiling for acute aneurysmal subarachnoid hemorrhage (SAH) between 2010 and 2020 were included (Fig. 1). The term “acute” refers to cases that presented within 14 days from the onset of the first symptoms. For exclusion criteria see Supplementary Table S1 online. All endovascular aneurysm treatments at our institution are performed under primary prophylactic heparin administration (see below for details). Two groups of intraprocedural antithrombotic medication were compared: Patients who received tirofiban for primary prophylactic use in addition to standard heparin administration (HEP + TF) versus patients who received only heparin (HEP) during coil embolization. Both groups also included cases of secondary antiplatelet agent use after completion of the coiling, e.g. due to a large coil surface at the level of the aneurysm neck, or cases of secondary antiplatelet agent use due to intraprocedural thrombus formation.

### Outcomes and data collection

Baseline patient and procedural information were sourced from digital medical records, intervention reports and anesthesia protocols. The CT and digital subtraction angiography (DSA) imaging conducted as part of the periprocedural diagnostics at that time were reviewed again in this analysis to extract image-related baseline and outcome data. The primary outcome event of our study was the occurrence of intraprocedural thromboembolic events. Thromboembolic events were defined as a lack of contrast filling of intracranial vessels during interventional DSA. Events were classified as (1) local thrombus formation in the parent artery adjacent to the aneurysm neck, (2) embolization into distal vessels or (3) proximal vessel occlusion. The secondary outcome of our study was the occurrence of ICH. Events of ICH were recorded from the onset of the intervention until 6 h after its completion, taking into account the effect duration of tirofiban. Cases of ICH were confirmed with cranial CT scans including intraprocedural flat-panel detector CT imaging or interventional DSA showing contrast extravasation. ICHs were further classified according to context (perforation, related to external ventricular drains (EVD), spontaneous i.e. non-mechanically provoked and distinct from the aneurysm) as well as clinical relevance based on the Heidelberg Bleeding Classification^13^. Accordingly, any new or progressive ICH during the specified period was defined as symptomatic if it resulted in clinical deterioration of the patient (without alternative explanations such as vasospasm or new ischemic infarction), as defined by^13^:

- The need for intubation, hemicraniectomy, (further) EVD insertion, other major medical or surgical procedures or.
- A sudden increase of ≥ 4 points in total National Institutes of Health Stroke Scale (NIHSS) score or ≥ 2 points in one NIHSS category or.
- Absence of an alternative explanation for deterioration.

Aneurysm size and morphology were assessed by experienced neurointerventionists (MW, CW) as described in^14^.

### Antithrombotic medication protocols

During the intervention, all patients underwent activated clotting time (ACT)-guided systemic heparinization via intermittent intravenous bolus administration of body weight-based heparin (target ACT ≥ 200 s). In the tirofiban (HEP + TF) group, patients additionally received an intravenous tirofiban loading dose of up to 0.4 µg/kg/min over 30 min adjusted to body weight and renal function. This was done either at the start of the intervention or at various timepoints during coil embolization (see below), followed by a 0.1 µg/kg/min maintenance infusion of tirofiban. The intraprocedural antithrombotic medication protocol, including the timing of primary prophylactic tirofiban administration, was at the discretion of the interventionist. Specifically, in 38 of 45 cases tirofiban was administered before insertion of the first coil or after insertion of the first or second coil. In the remaining 7 cases tirofiban was administered later than the third coil was inserted. In all 45 cases, tirofiban was administered before the aneurysm was angiographically occluded.

If secondary prophylactic use of antiplatelet agents was prescribed by the interventionist, 500 mg aspirin and/or tirofiban (according to the above dosing scheme) were administered intravenously. In the specific case of secondary tirofiban use for treatment of acute thrombus formation during the procedure, our standard is to intravenously administer a bolus of half the standard loading dose for immediate thrombolysis.

### Statistical analysis

Baseline nominal and ordinal data were presented as number (frequency) and median with interquartile range (IQR), respectively. Continuous data were presented as mean with standard deviation (SD) when distribution was normal and as median with IQR when distribution was not normal. Fisher’s exact tests were used to compare the primary and secondary endpoints between the HEP group and the HEP + TF group. To verify these results with regard to potential imbalances in receiving primary prophylactic tirofiban, we then conducted Cochrane-Mantel-Haenszel tests in a propensity score-matched subsample^15^. In accordance with current literature, the following covariates were included in the regression model to calculate propensity scores for each patient^14,16–19^: patient age, irregular aneurysm morphology, neck size, intracerebral bleeding on initial CT, pre-procedural Hunt and Hess grade, procedure time and delayed timing of treatment after SAH (> 36 h). We performed 1:2 matching using the greedy nearest neighbor matching method and a caliper width of 0.5 of the standard deviation of the logit of the estimated propensity score^20^. Covariate balance was considered good when the standardized mean difference (SMD) was ≤ 0.25 with a variance ratio between 0.5 and 2 ^21,22^. A chi-square test was conducted to compare the frequency of tirofiban administration among the interventionists.

Statistical analyses were conducted with SAS version 9.4 (SAS Institute, Cary, NC). Missing data are individually denoted in the results.

## Results

### Baseline characteristics

Of 739 aneurysm treatments from our single center registry data, 204 cases were included in the final analysis. There were 45 cases where primary prophylactic tirofiban was administered in addition to heparin (HEP + TF), and 159 cases where only heparin was administered as primary prophylaxis (HEP). The procedures during the specified period were performed by a total of 12 interventionists, six of whom had more than 5 years of interventional experience and six had between 2 and 5 years. More than 70% of the procedures were performed by the interventionists with more than 5 years of experience. Almost all interventionists used tirofiban with no significant differences between them, with rates ranging from 0 to 38% in the overall cohort (*p* = 0.051) and from 0 to 46% in the matched cohort (*p* = 0.35). In the HEP group, 26 of 159 patients received secondary tirofiban for emergency treatment of acute thrombus formation during the procedure (as described above). Eight of 159 patients received secondary tirofiban after the completion of coil embolization either as a bolus or for 12 h to prevent secondary thrombus formation (e.g. in cases of protruding coils or a broad interface between parent vessel and aneurysm neck), and 23 additional patients were started on aspirin for this purpose. Similarly, 11 of 45 patients from the HEP + TF group were started on aspirin after the completion of coil embolization. The baseline characteristics for the overall study cohort (before PSM) are summarized in online Supplementary Table S2, showing comparable age, aneurysm size, neck size as well as rates of irregular aneurysm morphology, delayed timing of treatment after SAH (> 36 h), and antithrombotic premedication. The HEP + TF group had lower Hunt and Hess grades (2 (1–3) vs. 3 (2–4)), a lower rate of pre-existing intracerebral bleedings (15.6% vs. 32.1%, SMD 0.4), and a lower rate of pre-procedural EVD (62.2% vs. 68.6%, SMD 0.3). The procedure time was longer for the HEP + TF group (3.6 (2.5–4.5) vs. 2.8 (2-3.8) hours, SMD 0.5). After PSM, satisfactory balance was achieved between groups (HEP: 76 cases, HEP + TF: 40 cases) for all baseline variables (Table 1). The patients in the propensity score-matched subsample were treated on average one day (IQR, 0–2) after the onset of symptoms.

**Table 1:** Baseline characteristics of groups (after propensity score matching). Values are depicted as number (%), mean ± SD or median (IQR).

Variable		HEP + TF (*n* = 40)	HEP (*n* = 76)	SMD	Variance ratio
Age (yrs)		53.7 ± 14.1	54.1 ± 14.2	0.03	1.0
Pre-procedural Hunt and Hess grade		2 (IQR, 1.5-3)	2 (IQR, 1–3)		
Pre-existing intracerebral bleeding		7 (17.5%)	15 (19.7%)	0.1	0.9
Antiplatelet premedication		2 (5.4%)§^1^	7 (10.3%)§^2^	0.2	0.6
Anticoagulant premedication		1 (2.5%)	2 (2.8%)*^2^	0.02	0.9
Pre-procedural EVD		25 (62.5%)	45 (59.2%)	0.1	1.0
Irregular morphology		23 (57.5%)	42 (55.3%)	0.05	1.0
Neck size (mm)		2.7 (IQR, 2.1–3.5)	2.5 (IQR, 2-3.5)	0.003	0.6
Aneurysm size (mm)		6.2 (IQR, 4-8.6)	5.8 (IQR, 4.2–8.8)	0.02	1.0
Procedure time (hours)		3.5 (IQR, 2.5–4.4)	3.0 (IQR, 2.5–4.1)	0.2	1.6
Delayed timing of treatment after SAH		11 (27.5%)	20 (26.3%)	0.03	1.0
Location					
1	Internal carotid artery	7 (17.5%)	12 (15.8%)		
2	Posterior communicating artery	5 (12.5%)	12 (15.8%)		
3	Anterior choroidal artery	0	1 (1.3%)		
4	Middle cerebral artery	1 (2.5%)	2 (2.6%)		
5	Anterior cerebral artery	0	2 (2.6%)		
6	Anterior communicating artery	19 (47.5%)	30 (39.5%)		
7	Vertebral artery	1 (2.5%)	3 (4%)		
8	Basilar artery	5 (12.5%)	10 (13.2%)		
9	Posterior cerebral artery	0	0		
10	Posterior inferior cerebellar artery	1 (2.5%)	4 (5.3%)		
11	Superior cerebellar artery	1 (2.5%)	0		
12	Anterior inferior cerebellar artery	0	0		

Bold numbers indicate meaningful imbalance between groups in baseline variables. Special characters indicate differing numbers of observations (n) due to missing values: §^1^ *n* = 37, §^2^ *n* = 68, *^2^ *n* = 71.

EVD, external ventricular drain; HEP, heparin; Procedure time, interval between first and last angiographic series; SAH, subarachnoid hemorrhage; SMD, absolute standardized mean difference; TF, tirofiban.

### Primary and secondary outcomes for the unmatched study groups

There were significantly fewer intraprocedural thromboembolic events under primary prophylactic tirofiban administration compared to the primary prophylactic administration of heparin only (HEP + TF, 2.2% vs. HEP, 22%, *p* = 0.0014). The hemorrhage rates did not differ between groups (all ICH: HEP + TF, 23.3% vs. HEP, 32.1%, *p* = 0.35; symptomatic ICH: HEP + TF, 4.7% vs. HEP, 7.1%, *p* = 0.74). The detailed results from the group comparison between HEP and HEP + TF among the overall study cohort are presented in online Supplementary Table S3.

### Primary outcomes for the propensity score-matched study groups

There were significantly fewer intraprocedural thromboembolic events under primary prophylactic tirofiban administration compared to the primary prophylactic administration of heparin only (OR 7.8 (95%CI 1 to 57.4); *p* = 0.017) (Table 2).

**Table 2:** Comparison of intraprocedural outcomes between matched groups. Values are depicted as number (%). Bold numbers indicate significant group differences as determined by Cochrane-Mantel-Haenszel statistics.

	HEP + TF (*n* = 40)	HEP (*n* = 76)	OR	95% CI	*P* value	
Thromboembolic events	1 (2.5%)	15 (19.7%)	7.8	1 to 57.4	**0.017**	
ICH	8 (20.5%)*	23 (30.7%)§	1.6	0.6 to 4	0.29	
Symptomatic ICH	2 (5.1%)*	3 (4%)§	0.9	0.1 to 5.5	0.88	

Special characters indicate differing numbers of observations (n) due to missing values: § *n* = 75, * *n* = 39.

HEP, heparin; ICH, intracranial hemorrhage; TF, tirofiban.

The 15 thromboembolic events in the HEP group comprised 9 cases of local thrombus formation adjacent to the aneurysm neck, one case of distal embolization and five cases of proximal vessel occlusion. Two cases required mechanical thrombectomy, while the remaining cases were treated with tirofiban. The only thromboembolic complication in the HEP + TF group was an incomplete occlusion of the right-sided acute middle cerebral artery (MCA) M2 segment observed at the end of coil embolization of an aneurysm at the origin of the right posterior communicating artery. The thrombus was extracted using a stent-retriever. Macroscopically the extracted thrombus appeared to consist of fibrin-rich older thrombotic material. The origin of the thromboembolism remained unclear. Follow-up imaging did not show evidence of an infarction.

### **Secondary outcomes for the propensity score-matched study groups**

Regarding ICHs and symptomatic ICHs, the intraprocedural incidence did not differ significantly between groups (all ICH: OR 1.6 (95% CI 0.6 to 4.0); *p* = 0.29 and symptomatic ICH: OR 0.9 (95% CI 0.1 to 5.5); *p* = 0.88) (Table 2). In the HEP + TF group, one symptomatic ICH occurred spontaneously while another was associated with intraprocedural aneurysm perforation. There were three symptomatic ICHs in the HEP group: Two occurred spontaneously, and one case in the context of external ventricular drainage. Only the symptomatic ICH related to aneurysm perforation was diagnosed intraprocedurally, while the remaining 4 cases were diagnosed postinterventionally during the 6-hour observation period.

Online Supplementary Table S4 provides detailed descriptive information on cases of symptomatic ICHs in both groups.

## Discussion

To the best of our knowledge, this study is the first to examine the primary prophylactic use of intravenous tirofiban during stand-alone coil embolization of ruptured cerebral aneurysms compared to treatment under prophylactic intravenous heparin (HEP) only. Patients under prophylactic tirofiban (TF) treatment suffered from significantly less thromboembolic events without an increased risk of intracranial hemorrhage (ICH) or symptomatic ICH.

In device-assisted aneurysm treatment, antiplatelet therapy (AT) is routinely applied to prevent thromboembolic complications and e.g. stent-thrombosis. For stand-alone coiling of ruptured aneurysms, systemic heparinization is standard of care. Additional AT is generally avoided due to the associated bleeding risk. The risk of thromboembolic events and related ischemic strokes in coil embolization is considerable ranging from 5 to 26%^16,18,23,24^, with various potentially thrombogenic sources such as the guiding catheter, intimal injury, protruding coils or migrating intra-aneurysmal thrombus material^1,25^. Our study confirms this relatively high rate of thromboembolic events for patients treated under heparin alone with 20%, whereas patients treated under additional tirofiban showed a rate of only 3%. Compared to a meta-analysis by Takase et al. of 410 ruptured aneurysms treated with stand-alone coiling under prophylactic antiplatelet therapy (aspirin or clopidogrel)^12^, our study results suggest an even lower thromboembolic event rate under tirofiban compared to aspirin and/or clopidogrel (3% vs. 10%). In contrast to the studies included in this meta-analysis, which primarily examined conventional antiplatelet agents, we focused on prophylactic tirofiban. This GP IIb/IIIa antagonist offers the advantages of reversible platelet aggregation inhibition and a short half-life, thus enabling better control of coagulation management in critically ill patients who may undergo further interventions (e.g. ventriculostomy or decompressive hemicraniectomy)^25,26^.

In the present study, a total of two cases of symptomatic ICH (5.1%) occurred under prophylactic tirofiban treatment (HEP + TF). One case was associated with aneurysm perforation. The other case showed progression of a pre-existing small hematoma. There was no case of aneurysm rebleeding in our study cohort following early intraprocedural administration of tirofiban. This might be attributable to the experimental and clinical observation that GP IIb/IIIa antagonists cause disaggregation of newly formed platelet aggregates but do not exert fibrinolytic effects^27^. These effects are considered to play a key role in the disaggregation of older clots^27–29^. Accordingly, no relevant dissolving effect of tirofiban would be expected on the thrombus, which seals the aneurysm rupture point after acute subarachnoid hemorrhage (SAH) followed by spontaneous hemostasis with fibrin nets at the time of treatment^28,30^.

We found that tirofiban did not increase the intracranial hemorrhage risk (HEP + TF: 21% vs. HEP: 31%). The relatively high overall bleeding rate in both groups from our study is most likely explained by inconsistent definitions of ICH. We recorded ICH events of every extent (including small local bleedings, e.g. ventriculostomy-associated), while others primarily defined hemorrhage as contrast agent extravasation (with AT: 11.9% vs. without AT: 11.5% )^12^. Following this definition, the rate of overall intracranial hemorrhage of our study cohort would be even lower, at 5.1% and 8% (HEP + TF vs. HEP).

During stand-alone coiling of ruptured aneurysms, tirofiban has been used for treating thromboembolic events and prophylactically for pre-defined indications. In a study by Liang et al.^31^, tirofiban was administered prophylactically to a subgroup of 46 patients with aneurysmal SAH, but this was done after satisfactory aneurysm obliteration, and only in the specific cases of coil protrusion, a large coil/parent artery interface, or suspected impairment of antegrade flow. They reported rates of ICH and thromboembolic events of 0% and 2.2%, respectively. However, the preprocedural risk profile was not reported for the stand-alone coiling subgroup, nor were any control group data, impeding a reasonable comparative interpretation of tirofiban application and dosing schemes in light of the absolute procedural complication rates from both studies. Moreover, considering that tirofiban was given post-coiling, tirofiban-related ICH in the context of the coiling procedure itself (i.e. aneurysm perforation) did not contribute to the bleeding rate in the mentioned subgroup.

## Limitations

There are some limitations to this study. During the postinterventional observation period, many patients remained intubated. In these patients, we may have missed potential cases of symptomatic ICH, which would have only become apparent in awake and fully neurologically evaluable patients. We focused on ICH related to intraprocedural tirofiban administration. Accordingly, no conclusions can be drawn regarding hemorrhagic complications in peripheral anatomic locations or prolonged tirofiban administration beyond the early postinterventional phase. No testing was done to confirm platelet response to tirofiban. However, tirofiban has previously been shown to act as a potent antiplatelet agent even in poor responders to aspirin and/or clopidogrel. During the embolization procedure, tirofiban was administered to different patients at varying timepoints, but always before achieving final aneurysm obliteration. Therefore, the efficacy and safety evaluation o^9,32,33^f prophylactic tirofiban within the framework of the present study refers only to ‘early’ administration in general. Further studies are required to determine the optimal timing of administration. This was a retrospective single-center analysis, and prescription of the antiplatelet regime was left to the decision of the treating interventionist. Propensity score matching was applied to eliminate potential selection bias, which was crucial given the initial group imbalances considering procedure time, rates of pre-existing intracerebral bleedings and pre-procedural EVD as well as pre-procedural Hunt and Hess grades. This approach can adjust for known patient characteristics, but unknown confounding factors can only be balanced in randomized controlled trials^34^. Consequently, the results of our study need to be confirmed in further studies with prospective and multicentric design.

## Conclusion

According to our study, prophylactic early intraprocedural tirofiban administration in addition to systemic heparinization seems to be safe and effectively lowers the rate of thromboembolic events in stand-alone coil embolization of acutely ruptured intracranial aneurysms. Primary prophylactic heparin treatment does not appear to be sufficient to prevent thromboembolic events in this context. Our findings suggest that tirofiban might lower thromboembolic event rates even more effectively than aspirin or clopidogrel. Further studies are warranted to confirm these results.

## Electronic supplementary material

Below is the link to the electronic supplementary material.

Supplementary Material 1

## Data Availability

Any additional information regarding our aneurysm database are available upon reasonable request. Please contact the Director of the Department of Diagnostic and Interventional Neuroradiology, Univ.-Prof. Dr. med. Martin Wiesmann (mwiesmann@ukaachen.de) to request access to the data.
