# Supplementary material for: Propensity score-adjusted analysis on early tirofiban administration to prevent thromboembolic complications during stand-alone coil embolization of ruptured aneurysms
Source: Sci Rep. 2024 Nov 1;14:26350. doi: 10.1038/s41598-024-77354-1 (PMC11530453; doi:10.1038/s41598-024-77354-1)
Supplement: Supplementary file 1 — Supplementary Material 1 [file 41598_2024_77354_MOESM1_ESM.pdf]

Propensity score-adjusted analysis on early tirofiban administration to prevent thromboembolic complications during stand-alone coil embolization of ruptured aneurysms

Authors: Franziska Bürkle[1], Charlotte S Weyland[1], Dimah Hasan[1], Farzaneh Yousefi[1], Hani Ridwan[1], Omid Nikoubashman[1], Martin Wiesmann[1*]

[1]Department of Diagnostic and Interventional Neuroradiology, University Hospital RWTH Aachen, Aachen, Germany

Corresponding author: Martin Wiesmann; mwiesmann@ukaachen.de

- extracranial or unruptured aneurysms
- alternative primary therapy to stand-alone coiling (parent vessel stenting, primary parent artery occlusion, intrasaccular aneurysm device, surgical clipping prior to coiling of aneurysm remnant)
- alternative primary prophylactic antithrombotic medication protocol (primary prophylactic aspirin use, administration of recombinant tissue plasminogen activator (rt-PA) prior to aneurysm coiling, single tirofiban bolus administration without maintenance dose)
- underlying condition with specific additional risk profile (arteriovenous malformations, carotid-cavernous fistulas, dissections)
- aneurysms from different study cohorts treated in one session

Supplemental Table S1. Exclusion criteria.

Variable		HEP+TF (n=45)	HEP (n=159)	SMD	Variance ratio
Age (yrs)		53.8 ± 13.8	57 ± 13.4	0.2	1.1
Pre-procedural Hunt and Hess grade		2 (IQR, 1-3)	3 (IQR, 2-4)		
Pre-existing intracerebral bleeding		7 (15.6 %)	51 (32.1 %)	**0.4**	0.6
Antiplatelet premedication		3 (7.5 %)§[1]	9 (6.9 %)§[2]	0.01	1.0
Anticoagulant premedication		2 (5 %)*[1]	6 (4.4 %)*[2]	0.2	1.3
Pre-procedural EVD		28 (62.2 %)	109 (68.6 %)	**0.3**	1.1
Irregular morphology		25 (55.6 %)	102 (64.2 %)	0.2	1.1
Neck size (mm)		2.7 (IQR, 2.1-3.5)	2.4 (IQR, 1.8-3.1)&	0.2	0.7
Aneurysm size (mm)		6.2 (IQR, 3.7-8.6)	5.9 (IQR, 4.3-8.7)&	0.1	1.3
Procedure time (hours)		3.6 (IQR, 2.5-4.5)#[1]	2.8 (IQR, 2-3.8)#[2]	**0.5**	1.4
Delayed timing of treatment after SAH		13 (28.9 %)	41 (25.8 %)	0.1	1.1
Location					
1	Internal carotid artery	9 (20 %)	25 (15.7 %)		
2	Posterior communicating artery	6 (13.3 %)	19 (12 %)		
3	Anterior choroidal artery	0	2 (1.3 %)		
4	Middle cerebral artery	1 (2.2 %)	4 (2.5 %)		
5	Anterior cerebral artery	1(2.2 %)	11 (6.9 %)		
6	Anterior communicating artery	20 (44.4 %)	73 (45.9 %)		
7	Vertebral artery	1 (2.2 %)	4 (2.5 %)		
8	Basilar artery	5 (11.1 %)	13 (8.2 %)		
9	Posterior cerebral artery	0	1 (0.6 %)		
10	Posterior inferior cerebellar artery	1 (2.2 %)	5 (3.1 %)		
11	Superior cerebellar artery	1 (2.2 %)	1 (0.6 %)		
12	Anterior inferior cerebellar artery	0	1 (0.6 %)		

Supplemental Table S2. Baseline characteristics for the overall study cohort. Values are depicted as number (%), mean ± SD or median (IQR). Bold numbers indicate meaningful imbalance between groups in baseline variables.

Special characters indicate differing numbers of observations (n) due to missing values:

§[1] n=40

§[2] n=131

*[1] n=40

*[2] n=135

& n=158

#[1] n=42

#[2] n=156

EVD, external ventricular drain; HEP, heparin; Procedure time, interval between first and last angiographic series; SAH, subarachnoid hemorrhage; SMD, absolute standardized mean difference; TF, tirofiban.

	HEP+TF (n=45)	HEP (n=159)	OR	95% CI	P value
Thromboembolic events	1 (2.2 %)	35 (22 %)	12.4	1.7 to 93.4	**0.0014**
ICH	10 (23.3 %)*1	50 (32.1 %)*2	1.6	0.7 to 3.4	0.35
Symptomatic ICH	2 (4.7 %)*1	11 (7.1 %)*2	1.6	0.3 to 7.3	0.74

Supplemental Table S3. Comparison of intraprocedural outcomes among the overall study cohort. Values are depicted as number (%). Bold numbers indicate significant group differences as determined by Fisher's exact test.

Special characters indicate differing numbers of observations (n) due to missing values:

*1 n=43

*2 n=156

HEP, heparin; ICH, intracranial hemorrhage; TF, tirofiban.

	Patient no	Age range	Pre-procedural H-H grade	Symptomatic ICH context (compartment)	Item leading to classification of new ICH as symptomatic*
HEP+TF	**1**	**30s**	**4**	**Perforation**	**Additional implantation of an EVD**
	**2**	**20s**	**4**	**Spontaneous (subdural)**	**Surgical hematoma evacuation**
HEP	**1**	**70s**	**5**	**Spontaneous (subarachnoid)**	**Tonsillar herniation; no alternative explanation**
	**2**	**50s**	**3**	**EVD**	**Decompressive hemicraniectomy**
	**3**	**60s**	**4**	**Spontaneous (intracerebral)**	**Grade 2 hemorrhage (PH2) within pre-existing infarct**
	4	70s	4	Spontaneous (intraventricular)	Sudden presentation with dilated, non-reactive pupils in the presence of massive rebleeding event; no alternative explanation
	5	60s	3	Spontaneous (intraventricular)	Implantation of an EVD
	6	60s	3	Perforation	Surgical hematoma evacuation
	7	50s	3	Spontaneous (subarachnoid)	Decompressive hemicraniectomy
	8	50s	4	Perforation	Decompressive hemicraniectomy
	9	60s	3	Spontaneous (subarachnoid and intracerebral)	Decompressive hemicraniectomy
	10	60s	4	Spontaneous (intracerebral and intraventricular)	Implantation of an EVD
	11	50s	3	Spontaneous (intracerebral)	Acute unresponsiveness in the presence of new mesencephal hemorrhage; no alternative explanation

Supplemental Table S4. Characteristics of patients with symptomatic ICH. Bold lines represent cases of symptomatic ICH that were included in the analysis after propensity score matching. The remaining lines represent cases of symptomatic ICH derived from the overall cohort.

*Based on the Heidelberg Bleeding Classification.

EVD, external ventricular drain; HEP, heparin; H-H grade, Hunt and Hess grade; ICH, intracranial hemorrhage; PH2, parenchymatous hematoma grade 2 according to the Heidelberg Bleeding Classification, defined as hematoma occupying 30 % or more of the infarcted tissue with obvious mass effect; TF, tirofiban.
